# The complete linkage disequilibrium test: a test that points to causative mutations underlying quantitative traits

**DOI:** 10.1186/1297-9686-43-20

**Published:** 2011-05-23

**Authors:** Eivind Uleberg, Theo HE Meuwissen

**Affiliations:** 1 Department of Animal and Aquacultural Sciences, Norwegian University of Life Sciences, 1432 Ås, Norway; 2 Norwegian Institute for Agricultural and Environmental Research, Arctic Agriculture and Land Use Division, 9269 Tromsø, Norway

## Abstract

**Background:**

Genetically, SNP that are in complete linkage disequilibrium with the causative SNP cannot be distinguished from the causative SNP. The Complete Linkage Disequilibrium (CLD) test presented here tests whether a SNP is in complete LD with the causative mutation or not. The performance of the CLD test is evaluated in 1000 simulated datasets.

**Methods:**

The CLD test consists of two steps i.e. analysis I and analysis II. Analysis I consists of an association analysis of the investigated region. The log-likelihood values from analysis I are next ranked in descending order and in analysis II the CLD test evaluates differences in log-likelihood ratios between the best and second best markers. Under the null-hypothesis distribution, the best SNP is in greater LD with the QTL than the second best, while under the alternative-CLD-hypothesis, the best SNP is alike-in-state with the QTL. To find a significance threshold, the test was also performed on data excluding the causative SNP. The 5^th^, 10^th ^and 50^th ^highest T_CLD _value from 1000 replicated analyses were used to control the type-I-error rate of the test at p = 0.005, p = 0.01 and p = 0.05, respectively.

**Results:**

In a situation where the QTL explained 48% of the phenotypic variance analysis I detected a QTL in 994 replicates (p = 0.001), where 972 were positioned in the correct QTL position. When the causative SNP was excluded from the analysis, 714 replicates detected evidence of a QTL (p = 0.001). In analysis II, the CLD test confirmed 280 causative SNP from 1000 simulations (p = 0.05), i.e. power was 28%. When the effect of the QTL was reduced by doubling the error variance, the power of the test reduced relatively little to 23%. When sequence data were used, the power of the test reduced to 16%. All SNP that were confirmed by the CLD test were positioned in the correct QTL position.

**Conclusions:**

The CLD test can provide evidence for a causative SNP, but its power may be low in situations with closely linked markers. In such situations, also functional evidence will be needed to definitely conclude whether the SNP is causative or not.

## Background

QTL mapping efforts often result in the detection of genomic regions that explain quantitative trait variation, but seldom in the detection of the causative mutation underlying the trait variation. Recently, methods developed to genotype high numbers of SNP have permitted to reduce the size of the genomic regions detected. High density SNP genotyping enables the detection of QTL regions of up to 2 cM in size. Availability of genome sequences and/or comparative maps make it possible to set up a shortlist of positional candidate genes. These candidate genes can be sequenced by second-generation sequencing technologies, leading to the detection of many potentially causative SNP that probably include the causative mutation. However, genetic approaches cannot distinguish between SNP in complete linkage disequilibrium (CLD) with the QTL and the QTL itself and at best, they can test whether a SNP is in complete LD with the QTL or not. Because false discovery rate and power are tightly connected when dealing with complex traits [1], the challenge is to find methods that provide sufficient power to discover a complete LD SNP and simultaneously keep the false discovery rate under control.

Recently, we investigated the effect of precision and power obtained by including the causative mutation among the markers in a QTL mapping experiment [2]. Both power and precision were increased and the results indicated that it would be possible to identify causative- or CLD-SNP. In this paper, we propose a test to identify SNP that are in complete LD with the QTL, in order to maximise the genetic evidence for the SNP that is the causative mutation. We evaluate the performance of this test using simulated data where the causative SNP is unequivocally known.

## Methods

### The simulated datasets

**Figure 1:**
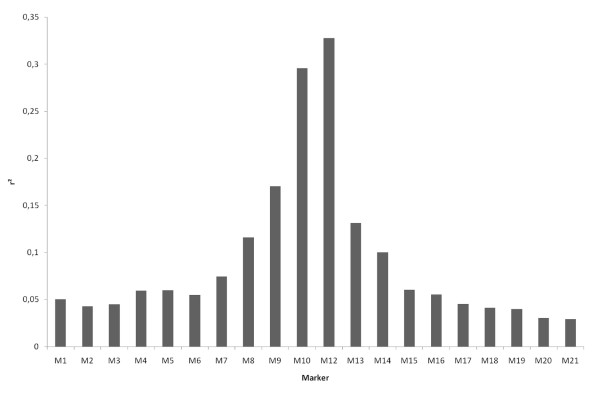
**Average r^2 ^between the causative and the 20 other SNP (from the first 100 replicates)**.

The simulated data used in this study have been previously described in Uleberg and Meuwissen [2]. Briefly, the SNP marker data were generated by Hudson's coalescence tree simulation program, "ms" [3] using a 2 cM long segment and 100 individuals (200 haplotypes). In practical situations, the size of the region depends on the confidence interval of the previous QTL mapping study. The assumed effective population size was 100, and the mutation rate was 10^-8 ^per bp (10^6 ^bp per cM was assumed). The size of the effective population did not exceed that of the sample, which is usually the case in livestock and which makes the continuous time approximation of the coalescence process somewhat unrealistic. In spite of this, we expected the resulting genealogies to resemble those in QTL mapping experiments involving unrelated individuals, such as Genome Wide Association Studies (GWAS). In addition to the 100 replications previously analysed by Uleberg and Meuwissen [2], 900 new replications were performed resulting in a total number of replicates of 1000. From the numerous markers generated by the "ms" simulations, 21 were selected based on position and allele frequency. The selected markers had minor allele frequencies (MAF) > 0.1 and were close to equidistant over the region, so that the average distance between two markers was 0.1 cM. The 11^th ^SNP was selected as the causative SNP and the effect of the QTL genotype was 0, 1 or 2. Phenotypic records were obtained by summing the QTL genotype effect and an environmental effect, which was sampled from N(0, 0.5). The average genetic variance (from the first 100 replicates) was 0.48, leading to a heritability of 0.54. Two datasets were selected for each of the 1000 simulations i.e. one containing 20 markers but not the causative SNP and one containing 21 markers including the causative SNP as the 11^th ^marker. Figure 1 shows the average linkage disequilibrium measured by r^2 ^[4] between the causative SNP and the other 20 SNP as a function of their distance to the causative SNP.

### Statistical analysis

The analysis consisted of two steps: analysis I and analysis II.

In analysis I, a QTL analysis of the region was performed using a statistical model that regressed directly on marker effects, as in association mapping, calculating the log-likelihood of effects of the different markers. The model assumed additive inheritance and was:

where μ is an overall mean, **1 **is a vector of ones, **m **is a vector of two random SNP allelic effects and **e **is a vector of random sampling errors; **Z **is a design matrix indicating which marker alleles are carried by the animals. The correlation matrix of **m **is the identity matrix, **I**. The variance of the random effects **m **and **e **and the log-likelihood of the model were estimated using the ASREML package [5]. A model containing the marker alleles was tested against a model excluding the marker alleles. The log-likelihood ratio, i.e. the difference of log-likelihoods between the two models, was used as a criterion for evidence of a QTL at the putative marker position. Next, the SNP were ranked for their log-likelihood values, where the most likely SNP was denoted (1), the second most likely (2), etc.

In Analysis II, the two SNP that gave the highest log-likelihood values in analysis I were compared by the CLD test. The idea is that, if the maximum-likelihood-SNP is in complete LD with the QTL, it will not only have a high Identity-By-Descent (IBD) probability with the QTL but also be alike-in-state (AIS) and thus will explain substantially more variance than a SNP that is only in partial LD with the QTL, such as the second highest log-likelihood SNP. The test statistic is thus:

where LogLik(m_(i)_) is the log-likelihood of the model including the *i*-th ranking marker. The T_CLD _values are a measure of the relative importance of the best SNP compared to the second best SNP. Since the best SNP is expected to explain more variance than the second best SNP, the null-hypothesis distribution differed from the usual one, i.e. the best SNP was expected to explain more variance. Thus, under the null-hypothesis distribution, the best SNP is in somewhat more LD with the QTL than the second best SNP, whereas under the alternative-hypothesis the best SNP is in complete LD with the causative mutation and thus also alike-in-state with the QTL. In order to establish a significance threshold for the CLD test, the test was also performed on data where the causative SNP was excluded. The 5^th^, 10^th ^and 50^th ^highest T_CLD _value out of 1000 replicated analyses excluding the causative SNP were taken as the p = 0.005, p = 0.01 and p = 0.05 significance threshold, respectively.

## Results

**Figure 2:**
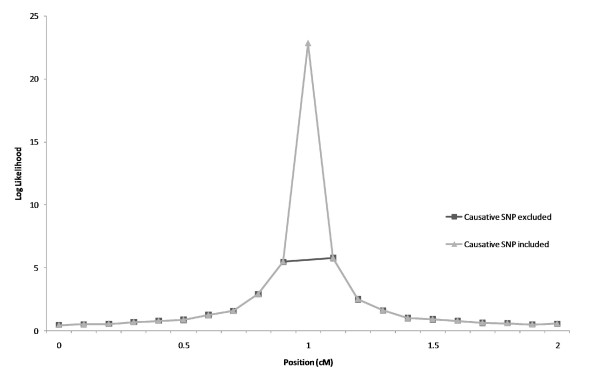
**Average log-likelihood ratios for 1000 simulations when the causative SNP was included or excluded from the analysis**.

Figure 2 shows mean log-likelihood ratio values from the analysis including or excluding the causative SNP. The average log-likelihood ratio for the most likely SNP position was ~ 6 when the causative SNP was excluded and ~ 23 when the causative SNP was included. Based on 100 replicates, the LD, measured by r^2^, was 0.33 between the QTN and the best adjacent marker. The average r^2 ^between all markers was 0.2.

### Analysis I: causative SNP included

Table 1 shows that analysis I detected a QTL in 994 replicates (p = 0.001) when the causative SNP was included in the analysis. In 972 replicates, the detected QTL was positioned in the 11^th ^marker position, which was the correct position.

**Table 1:** Precision of QTL position estimates in 1000 replicate simulations

Original size of QTL								
**Number of brackets or marker positions between estimated and correct position (P = 0.001)**								
	**0**	**1**	**2**	**3**	**4**	**5**	**> 5**	**No significant QTL found**

QTL between markers*		447	132	59	26	22	28	286
**QTL included as marker**	972	14	4	3		1		6

**Reduced size of QTL**								

**Number of brackets or marker positions between estimated and correct position (P = 0.001)**								
	**0**	**1**	**2**	**3**	**4**	**5**	**> 5**	**No significant QTL found**

QTL between markers*		344	84	32	11	13	21	495
**QTL included as marker**	855	27	9	6		1	1	101

*since the QTL is not included there is no correct position; the two marker positions surrounding the QTL are considered to be one position away from the correct position

For 59 of the replicates, the best log-likelihood value was shared between two SNP. In 58 cases, this was the causative SNP and a SNP positioned 1-3 positions away from the causative SNP. For the 58 replicates when the causative SNP was amongst the SNP with equal log-likelihood values, the replicate was defined as correctly positioned in Table 1. The 59 simulations that found equal log-likelihood values for two SNP positions were not included in analysis II, because our ultimate aim was to find evidence for the causal SNP, and in these 59 cases, the genetic evidence is clearly inconclusive and more data is needed. The six replicates that did not find evidence of a QTL were also excluded from analysis II.

### Analysis I: causative SNP excluded

When the causative SNP was excluded from the analysis, evidence for a QTL at p = 0.001 was detected for 714 replicates. Four hundred and forty-seven of these were positioned adjacent to the masked causative SNP. The results from the first 100 simulations of analysis I have been presented by Uleberg and Meuwissen [2].

### Analysis II

**Figure 3:**
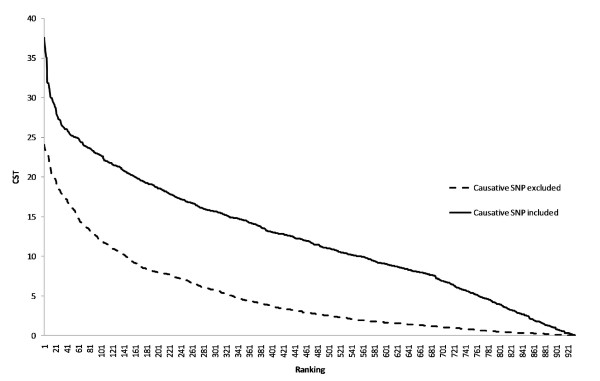
**T_CLD _test statistics when the causative SNP was included or excluded in the analysis**. T_CLD _values are ranked in descending order

Figure 3 shows the distribution of the T_CLD _values for the analysis when the causative SNP was included or excluded. T_CLD _values were generally higher when the causative SNP was included. The average T_CLD _value was 4.84 when the causative SNP was excluded and 12.45 when the causative SNP was included.

Table 2 shows that in analysis II, the CLD test confirmed 88 causative SNP for the 1000 simulations when the significance level was p = 0.005. When the significance level was reduced to p = 0.05, the CLD test confirmed 280 causative SNP. All confirmed SNP were positioned correctly by the initial analysis I. Thus, none of the 22 significant SNP that were not correctly positioned was confirmed by the CLD test. It should be noted that the CLD test involves only a single test for the entire segment, such that higher p-value thresholds can be used than when testing every SNP individually, and performing 21 tests.

**Table 2:** Power of the CLD test based on the number of significant associations in 1000 simulations for three threshold levels

Original size of QTL		
Significance threshold		
p = 0.005	p = 0.01	p = 0.05

**88**	**121**	**280**

**Reduced size of QTL**		

Significance threshold		
p = 0.005	p = 0.01	p = 0.05

**48**	**99**	**231**

### Effect of decreasing the size of the QTL

Additional analyses were performed to investigate the behaviour of the CLD test when the QTL effect size was reduced. The relative effect of the QTL was reduced by doubling the error variance from 0.5 to 1. In analysis I, the reduced QTL effect led to a decrease in average log-likelihood values for the most likely QTL position from ~ 6 to ~ 3 when the causative SNP was excluded from the analysis and from ~ 23 to ~ 12 when the causative SNP was included in the analysis. The number of detected causative SNP was reduced from 972 to 899 (Table 1). Eight hundred and fifty-five of the detected SNP were positioned at the position of the causative SNP. For 49 of the replicates, the best log-likelihood value was shared between two SNP. Again, these replicates were excluded from analysis II, as the evidence for a causative mutation was not conclusive. The 101 replicates that did not find evidence of a QTL were also excluded from analysis II.

Table 1 also shows that when the causative SNP was excluded from analysis I, the number of replicates that detected evidence for a causative SNP was reduced from 714 to 505 when the QTL effect was reduced. The results from the first 100 simulations of analysis I have been presented by Uleberg and Meuwissen [2].

**Figure 4:**
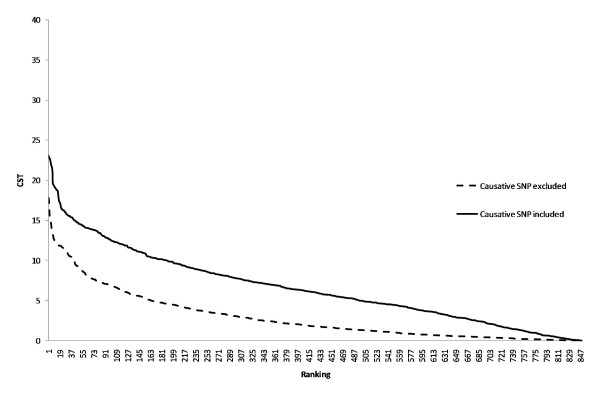
**T_CLD _test statistics when the causative SNP was included or excluded in the analysis and the QTL effect was reduced**. T_CLD _values are ranked in descending order

Figure 4 shows that, when the size of the QTL effect was reduced, the average T_CLD _values were reduced from 4.84 to 2.91 if the causative SNP was excluded and from 12.45 to 6.66 if it was included. Table 2 shows that, with a reduced QTL effect, the CLD test confirmed fewer causative SNP from the 1000 simulations. The number of confirmed causative SNP was reduced from 88 to 48 with a significance level of p = 0.005 and from 280 to 231 with a significance level of p = 0.05. Again, the position of all confirmed SNP was the same as that of the causative SNP determined by the initial analysis I.

### Relationship between T_CLD _values and marker statistics

We investigated the relationship between the T_CLD _value and the LD between the best and second best SNP: for the 50 highest T_CLD _values, the average r^2 ^was 0.23 and for the 50 lowest it was 0.85. This shows that a low r^2 ^between the best and second best SNP favours a high test statistic and thus produces a significant result. Thus, if the causative SNP is among the SNP being tested, there is a greater chance to obtain a positive result if the r^2 ^value between adjacent SNP is low and thus if marker density is low. The datasets with the 50 highest and 50 lowest T_CLD _values had an average minor allele frequency (MAF) for the causative SNP of 0.38 and 0.24, respectively, indicating that a higher MAF value favours a significant test result, although this effect is relatively small.

## Discussion

The proposed CLD test confirmed 280 out of 1000 causal SNP at a p-value of 0.05 (231 when the QTL effect size was reduced). The power of the CLD test is thus 23-28% and is much lower than when the SNP are used to detect QTL-SNP associations. This relatively low power reflects the fact that proving that a SNP is in complete LD is more difficult than showing that it is merely associated with the QTL. Thus, as previously reported [1], avoiding false discoveries results in lower power when trying to confirm causal SNP. Reducing the size of the QTL effect did not affect dramatically the power of the test, indicating that other factors, such as the LD structure in the region, are more important to the power of the test. The stringent threshold for the CLD test is the result of strong LD between the SNP in these data. Thus, the CLD test accounts for the background LD when trying to distinguish complete LD from associated SNP.

An alternative approach to find the causative SNP is the concordance test [6] in which the candidate SNP are genotyped in the parents of the families involved in the linkage mapping design. For this test, the QTL genotypes of the parents should be based on many offspring and be quite certain. If the SNP genotypes agree with that of the inferred QTL genotypes, it provides evidence for the SNP being causative. However, if a SNP is in strong LD with the QTL, the SNP genotypes are also expected to agree with the QTL genotypes, especially when there are only a few parents with 'almost' certain QTL genotypes. For example, in a coat colour mapping study in dogs, 37% of the candidate genes past the concordance test [7]. Moreover, if some of the QTL genotypes are wrongly inferred, this test results in a type-I-error [8]. The data used in this paper did not have the structure of a linkage mapping study, and thus QTL genotypes could not be inferred with high accuracy. The current data resembled that of an association study and, thus, the presented approach is suited to follow-up upon GWAS results.

The test-statistic of the CLD test is based on the assumption that, under the null-hypothesis distribution, the best SNP explains more variance of the phenotype than the second best, whereas under the alternative hypothesis the best SNP is also alike-in-state with the QTL and explains much more of the phenotypic variance. Based on the average log-likelihood values for all 1000 simulations, the difference between the best and second best SNP in these data is ~17 log-likelihood units when the causative SNP is included and only ~0.5 log-likelihood units when the causative SNP is excluded from the analysis. However, the variance between replicates is large, leading to a relatively low power when all replicates are evaluated.

In GWAS, isolated significant SNP are often distrusted, because none of the neighboring SNP confirms the presence of a QTL. In such a case of an isolated significant SNP, the CLD test would provide a positive result since its signal is so much higher than that of neighboring SNP. Here, we assumed that the previous QTL mapping study unequivocally detected a QTL in the studied region, so that regions with spurious significant SNP will not be subjected to the test.

QTL mapping cannot distinguish between a causative SNP and a SNP that is in perfect LD with the causative SNP [9]. Thus, if two SNP are found with equally high log-likelihood values, it is not clear which of the SNP is the causative mutation, and the CLD test statistic would be zero and should not be performed. The latter effect of having a low CLD statistic if one or more SNP are in very high LD with the causative SNP appears to protect the CLD test from pointing to non-causative SNP when the causative SNP is included in the analysis. This is demonstrated by the result that none of the 22 and 44 incorrectly positioned significant SNP in Table 1 are confirmed by the CLD test.

Since higher T_CLD_-statistics were found for SNP with a low r^2 ^with their nearest marker, we investigated the effect of SNP density on the power of the test. Here, we considered the highest possible density, namely sequence data, which is becoming increasingly available. We reran 1000 "ms"-simulations as described in the Methods section, but retained all the SNP that resulted from the simulated mutations. This resulted in an average of 470 SNP in the 2 cM segment, with an average r^2 ^between adjacent markers of 0.12. The average r^2 ^was rather low due to the often low MAF, but for 6% of the marker pairs r^2 ^was equal to 1. The SNP closest to the middle of the 2 cM segment was designated as the QTL and an environmental effect sampled from N(0,0.5) was added to obtain phenotypes. Out of 1000 replicates, 545 had a single most significant QTL, and 402 of these had the QTL correctly identified. Out of these 402 replicates, 63 had a significant T_CLD _statistic (P < 0.05), resulting in a power of 16% (= 63/402). Thus, the power was substantially reduced if the marker density was increased to that of sequence data, but some level of power remained. Again none of the misplaced QTL positions passed the CLD test.

The fact that high marker densities, such as in sequence data, results in a reduction of the power of the test, may suggest that removing some SNP from the data (obviously not the putative causative SNP) will improve power. However this invalidates the CLD test, since the test assumes that some SNP from the QTL region were obtained through a SNP discovery process that is not related to the phenotypic data. Moreover, this artificial reduction of SNP density can result in false positive test results, because the T_CLD _statistic will be artificially increased if the second best SNP is removed and, e.g., replaced by the *i*-th best.

In 59 replicates, analysis I found two or more SNP with equal log-likelihood values for the most likely SNP. This was typically the causative SNP and a SNP located 1 to 3 positions away from the causative SNP. Evaluating five of these replicates showed equal haplotype combinations for every animal for the two most likely SNP, thus the two SNP were in perfect LD. Other replicates produced similar results, with the causative SNP and one close SNP returning log-likelihood values at a higher level than the rest of the SNP, although not equal. In these replicates, the analysis excluding the causative SNP returned large T_CLD _values and resulted in the stringent significance threshold that was used here.

As explained by Goddard and Hayes [10], causative SNP might be expected to show different properties to common SNP, because causative SNP may be subject to selection such that polymorphisms will typically be recent and have low minor allele frequencies. Thus they may show less LD with markers than common SNP. As a consequence, causative SNP may be expected to show less LD to common SNP in real data than in these simulations, which may improve the power of the CLD test in real data, if the causative SNP is included. However, since we tend to choose common markers for SNP genotyping experiments, the causative SNP will less likely be included in real data as long as selection is based on the minor allele frequency. Hence, all SNP in the promising regions will have to be genotyped in order to improve the probability of inclusion of the causative SNP.

When SNP are evaluated, a number of these will be coding SNP that change amino acids [9]. The number of coding SNP is substantially smaller than the overall total number of common SNP. So far little effort has been placed on identifying coding SNP, but for the future, knowledge on which SNP are coding could be valuable when trying to identify causative mutations. Information about coding SNP will reduce the number of candidate SNP and thus improve the power of tests for causal SNP by removing the signal from non-coding SNP in LD with the causative SNP. However, non-coding SNP in regulatory regions of the genes may also be causative. If the candidate region contains several genes, information on gene function could also be used to increase the power of the test.

Including the causative SNP as a marker increased the average log-likelihood values about four times in these simulations (Figure 2). Although these simulations were quite simple, this large increase appears to be quite general, although its size may be modified by different factors, such as family structure, marker density, dataset size and QTL effect sizes. Given our general conclusion that the inclusion of the causative SNP is expected to increase the log-likelihood ratios, these factors are expected to affect mainly the power of the test.

To apply the CLD test to real data, the significance threshold must be estimated from the real data. The basic approach that is proposed is to perform a QTL analysis (i.e. analysis I), and to calculate the T_CLD _statistic (T_CLD(real)_). Then, records are simulated assuming that the SNP detected by the QTL analysis is causative, with simulated QTL variances equal to the estimates obtained from the real data analysis, where every SNP *i *will in turn be assigned as causative, and will be masked when analysing the data. This simulates replicated data under the null-hypothesis with an LD structure as found in the QTL region. Analysing these null-hypothesis data without including the assumed causative SNP will provide a significance threshold for the analysed data. A significance level can be obtained by counting how many of the null-hypothesis T_CLD _values exceed the real data T_CLD(real) _-value. For example, if 100 out of 1000 null-hypothesis datasets have T_CLD _values exceeding T_CLD(real)_, the p-value of the real data CST is 0.1 (= 100/1000).

The relatively low power of the CLD test does not imply that it should not be used, since it is not very costly to perform and, depending on its p-value, it may provide substantial statistical evidence for a causative SNP. However, because of the low power of the test, the p-value of the real data T_CLD _(as described in the previous paragraph) will in most situations be quite high. Ron and Weller [6] suggested that the quest for the causative SNP had to be won on points rather than by knockout. Their criteria for validating causality included linkage analysis and LD mapping, positional cloning, selection of candidate genes, DNA sequencing, and statistical analysis. Their conclusion was that only an array of evidence can establish proof of causality. The critical test will be concordance and functional validation. In this setting, the CLD test may provide considerable evidence for a causative SNP, especially when a concordance test cannot be applied, but due to its high p-value, functional evidence will be needed to definitely conclude whether the SNP is causative or not.

## Competing interests

The authors declare that they have no competing interests.

## Authors' contributions

EU carried out data analysis and drafted the manuscript. THEM participated in the design of the study and statistical analysis and helped draft the manuscript.

Both authors have read and approved the final manuscript.
